# 2299. Genomic Epidemiology of *SARS-CoV-2* in Metropolitan Detroit

**DOI:** 10.1093/ofid/ofad500.1921

**Published:** 2023-11-27

**Authors:** Jagjeet Kaur, Katherine Gurdziel, Benjamin Wasinski, Nivisa Vakeesan, Sayed Hassan Raza, Wanqing Liu, Marcus Zervos, Geehan Suleyman

**Affiliations:** Henry Ford Health, Detroit, Michigan; Wayne State University, Detroit, Michigan; Wayne State University, Detroit, Michigan; Wayne State University, Detroit, Michigan; Wayne State University, Detroit, Michigan; Wayne State University, Detroit, Michigan; Henry Ford Hospital, Detroit, Michigan; Henry Ford Health, Detroit, Michigan

## Abstract

**Background:**

The COVID-19 pandemic, resulting from the rapidly evolving SARS-CoV-2 virus, has drastically impacted health systems and economies worldwide. Genomic sequencing is critical for the surveillance of SARS-CoV-2 to monitor the rapidly evolving virus and identify new strains.

**Methods:**

583 isolates from Henry Ford Health were retrospectively profiled across 3 years (117 isolates in 2020; 39 in 2021; 427 in 2022). DNA was extracted using Kingfisher viral isolation kit; RT-PCR screening was used to identify isolates with cycle threshold < 32 for whole genome sequencing (WGS). Libraries were generated using QIAseq DIRECT SARS-CoV-2 Kit, followed by Illumina sequencing (MiSeq or NovaSeq 6000; 300 cycles). Lineage analysis of the SARS-CoV-2 consensus genome sequences generated from samtools variant analysis pipeline was determined using Nextclade and Pangolin software.

**Results:**

Sequences were classified into 11 unique clades across 108 lineages by Nextclade and Pangolin, respectively. Almost three-fourths of the sequenced isolates were from 2022. 117 (20%) genomes were from the early pandemic (2020) and were clustered into 8 clades: 19A, 19B, 20A, 20B, 20C, 21 J (Delta) and 21L (Omicron). Most of the 2022 genomes (72%) were in clade 20C from lineage B.1, identified during the outbreak in Europe. 15 (13%) genomes had clade 19A (lineage B) and 1 had 19B (lineage A.3), identified early in the pandemic in Wuhan, China. Of the 39 (7%) genomes from 2021, the majority (82%) clustered into clade Delta 21J that originated in India. Only 2 (5%) of the genomes from 2021 had Alpha variant of concern (21I) from the lineage B1.1.7, which were suspected to be more transmissible. Of the 427 (73%) genomes from 2022, 393 (92%) had variants within Omicron variant of concern (21L), with 79 different lineages; 1 was Omicron variant 21K from the lineage BA1.1. Other clades observed in the 2022 batch were 19A (6%), 20A (0.2%), 20B (0.2%), 20C (0.2%) and 21M (1%).

**Conclusion:**

Our genomic surveillance data suggest that SARS-CoV-2 infections at the local level mirrored global outbreaks. This underscores the importance of robust genomic surveillance efforts to inform public health planning and practice.

**Disclosures:**

**Marcus Zervos, MD**, Contrafect: Advisor/Consultant|GSK: Grant/Research Support|Johnson and Johnson: Grant/Research Support|Pfizer: Grant/Research Support

